# The Injection of Air/Oxygen Bubble into the Anterior Chamber of Rabbits as a Treatment for Hyphema in Patients with Sickle Cell Disease

**DOI:** 10.1155/2014/696302

**Published:** 2014-04-07

**Authors:** Emre Ayintap, Uğurcan Keskin, Fariz Sadigov, Mesut Coskun, Nilufer İlhan, Sedat Motor, Hilal Semiz, Nihan Parlakfikirer

**Affiliations:** ^1^Department of Ophthalmology, Bezmialem Vakif University, 34093 Istanbul, Turkey; ^2^Department of Ophthalmology, Mustafa Kemal University, 31000 Hatay, Turkey; ^3^Department of Biochemistry, Mustafa Kemal University, Hatay, Turkey

## Abstract

*Purpose.* To investigate the changes of partial oxygen pressure (PaO_2_) in aqueous humour after injecting air or oxygen bubble into the anterior chamber in sickle cell hyphema. *Methods.* Blood samples were taken from the same patient with sickle cell disease. Thirty-two rabbits were divided into 4 groups. In group 1 (*n* = 8), there was no injection. Only blood injection constituted group 2 (*n* = 8), both blood and air bubble injection constituted group 3 (*n* = 8), and both blood and oxygen bubble injection constituted group 4 (*n* = 8). *Results.* The PaO_2_ in the aqueous humour after 10 hours from the injections was 78.45 ± 9.9 mmHg (Mean ± SD) for group 1, 73.97 ± 8.86 mmHg for group 2, 123.35 ± 13.6 mmHg for group 3, and 306.47 ± 16.5 mmHg for group 4. There was statistically significant difference between group 1 and group 2, when compared with group 3 and group 4. *Conclusions.* PaO_2_ in aqueous humour was increased after injecting air or oxygen bubble into the anterior chamber. We offer to leave an air bubble in the anterior chamber of patients with sickle cell hemoglobinopathies and hyphema undergoing an anterior chamber washout.

## 1. Introduction

Hyphema can occur after blunt trauma, intraocular surgery, or spontaneously. The treatment of hyphema is difficult and needs to be performed carefully when it occurs in patients who have sickle cell hemoglobinopathies. In these particular groups of patients, hyphema may have devastating effects on the eye [1, 2]. Therefore, any treatment modality that may contribute to the treatment of this situation warrants consideration.

Sickle cell trait has high prevalence in some regions of the world. The prevalence of this disease in the US black population is 6.7–10.1% [1, 3]. Moreover, equatorial Africa, the Mediterranean, and the Middle East have the highest prevalence [3]. Therefore, sickle cell trait should be kept in mind in any case of hyphema encountered in these regions.

Low oxygen concentrations and low pH, as well as high ascorbate and carbon dioxide levels, can lead to increased sickling in the anterior chamber, thereby increasing the rate of complications due to hyphema [4, 5]. The risk of permanent vision loss due to hyphema in humans with sickle cell trait is relatively high [1, 6]. This occurs as a result of sickling of the erythrocytes, which slow down the ocular microcirculation [7]. As reported, in this group of patients, even a small amount of hyphema can cause IOP elevation, leading to central retinal artery occlusion [8]. Experiments have revealed that if the blood of a human with sickle cell trait and the blood of a normal human are injected into the anterior chamber of the rabbits, in the sickle cell trait group, hyphema lasts longer with higher IOP readings when compared with the normal human blood injected group [9].

It was found that some drugs used in the treatment of glaucoma in sickle cell disease/trait patients can aggravate sickling [1]. The sickled erythrocytes obstruct the trabecular meshwork, thereby decreasing the outflow of aqueous humor and increasing IOP. Different treatment modalities have been proposed to break this vicious cycle and take control of the IOP elevation. Hyperbaric oxygen therapy and transcorneal oxygen therapy are two examples of these treatment modalities [6, 10]. The main aim of these treatment strategies is to increase oxygen concentration in the anterior chamber in order to move sickled erythrocytes away from the trabecular meshwork. In transcorneal oxygen therapy, IOP increase, secondary to sickle cell hyphema, was successfully reduced [10]. In an animal model, it was shown that hyperbaric oxygen therapy increases the anterior chamber oxygen concentration and decreases the percentage of sickled erythrocytes [6].

The aim of this study is to investigate the changes of partial oxygen pressure (PaO_2_) in aqueous humour after injecting air or oxygen bubble into the anterior chamber in sickle cell hyphema. It is obvious that increasing oxygen concentration in the anterior chamber will decrease sickling of erythrocytes and will facilitate the clearance of sickled erythrocytes in trabecular meshwork.

## 2. Methods

This study was approved by the Animal Ethics Committee of Mustafa Kemal University and met the criteria suggested by the Association for Research in Vision and Ophthalmology.

Anaesthesia was performed using intramuscular ketamine hydrochloride (50 mg/kg) and xylocaine (4 mg/kg). During injection into the anterior chamber or sampling aqueous humour, proparacaine 0.5% was used as a topical anaesthetic. Thirty-two New Zealand rabbits weighing 2-3 kg were enrolled in the study. Only right eyes of the animals were used. Blood sample was taken from the same patient with sickle cell disease for injection into the anterior chamber. Similar to a previous study, after collection by venipuncture, the donor blood was immediately anticoagulated with EDTA and maintained at room temperature until being injected into rabbit anterior chambers within 1 hour [6].

All the animals were divided into four groups. No intracameral injection of gas or air was performed in group 1 (*n* = 8) and the oxygen concentration in the sampled aqueous humour was considered a baseline value. Animals in group 2 (*n* = 8) were injected intracamerally with the blood (0.1 cc) of a patient with sickle cell disease. Animals in group 3 (*n* = 8) were injected intracamerally with both the blood (0.1 cc) of a patient with sickle cell disease and an air bubble (0.05 cc). Animals in group 4 (*n* = 8) were injected intracamerally with the blood (0.1 cc) of a patient with sickle cell disease and oxygen bubble (0.05 cc). The concentration of oxygen bubble was 50%. A 3-way IV stopcock was placed between the injector and cannula. The oxygen line was placed to one side of the 3-way IV stopcock to enable injection 3 of the oxygen bubble to the anterior chamber. The blood and the air bubble were injected intracamerally via the limbus using a 30-gauge needle. In order to prevent air loss during air or oxygen injection, the eye was deviated to the site of the injection, thus making the bubble move away from the injection site. After removing the needle, a cotton bud was used for tamponade of the injection site.

Aqueous humour samples were taken with paracentesis after 10 hours from the injections. The paracentesis was performed using a 0.165 mL heparinized capillary tube with a fixed 30-gauge needle. The needle was inserted directly through the corneal limbus into the anterior chamber until the capillary tube was fully filled. Measurements were carried out using an arterial blood-gas analyser (GEM premier 3500, Instrumentation Laboratory, UK). All the animals were kept under observation for 4 weeks and followed up to see if they developed cataract or corneal edema.

Statistical analyses were performed using Statistical Package for the Social Sciences (SPSS) 16.0 Package (SPSS Inc., Chicago, IL, USA). Descriptive statistics were presented as mean ± standard deviation. The Kruskal-Wallis test was used to determine whether a significant difference was present among the 4 groups of animals. If a significant difference was observed, then the Mann Whitney* U* test was used to carry out pairwise comparisons between the individual groups.

## 3. Results

In the groups where air or oxygen gas was used (groups 3 and 4), the bubbles were averagely absorbed 8 hours following the injections. The PaO_2_ measurements of the aqueous humour at the tenth hour after injections were as follows: 78.45 ± 9.9 mmHg (mean ± standard deviation (SD)) in group 1, 73.97 ± 8.86 mmHg in group 2, 123.35 ± 13.6 mmHg in group 3, and 306.47 ± 16.5 mmHg in group 4 (Table 1).

There was no statistically significant difference between group 1 and group 2, but when these groups were compared with group 3 and group 4, a statistically significant difference was found. Moreover, there was a statistically significant difference between group 3 and group 4. Comparative statistical results between the groups are given in Table 2.

All the animals were examined on day 1, week 1, and week 4, and they were observed in terms of the development of cataract or corneal edema. In the 4th week, there were no side effects observed in the groups 1, 2, and 3. However, one cataract formation and one persistent corneal edema were observed in two separate rabbits of the group 4 at the end of this period.

## 4. Discussion

Elevated IOP in patients with sickle cell trait can cause serious problems. Central retinal artery occlusion was reported in young patients with sickle cell trait with even a small amount of hyphema presence [8]. As antiglaucomatous drugs can increase sickling, it is important to choose an appropriate drug in the management of IOP. Walton et al. stated that timolol can be started as a topical antiglaucomatous agent as a first step [1]. If a second antiglaucomatous agent is needed, they advise adding brimonidine or apraclonidine. If the IOP is still not in the safe range, then before starting oral carbonic anhydrase inhibitors, topical anhydrase inhibitor should first be administered [1]. A surgical intervention should be planned if medical treatment fails. The IOP baseline levels considered for glaucoma surgery in normal patients should be lowered in patients with sickle cell trait since they are more sensitive to high IOP levels. It has been reported that if the elevation of IOP is managed within 24 hours, a better prognosis is expected, whereas uncontrolled IOP during first 24 hours is likely to result in a more difficult treatment period [11]. In the same study, the authors recommend surgery for hyphema in patients with sickle cell trait after 24 hours if the average IOP remains 25 mmHg or more or if it increases transiently and repeatedly above 30 mmHg [11]. If we consider that sickle cell patients are more sensitive to high IOP levels, then early surgical intervention will be beneficial.

Hyperbaric and transcorneal oxygen treatments have been proposed as the treatment for IOP elevation due to hyphema in patients with sickle cell disease/trait [6, 10]. It has been reported that hyperbaric oxygen treatment following the intracameral injection of blood of patients with sickle cell trait decreased sickling of the cells and increased the PaO_2_ in the anterior chamber [6]. Another study revealed that transcorneal oxygen given through modified goggles lowers IOP [10]. The main purpose of these treatments is to increase PaO_2_ in the anterior chamber by decreasing the number of sickled cells, thereby increasing their washout process from the anterior chamber through the trabecular pathway, and decrease IOP.

In our study, we found a statistically significant increase in PaO_2_ of the aqueous humour following the intracameral injection of air or oxygen bubbles. Previous studies found different baseline levels of PaO_2_ in rabbit eyes ranging from 30.5 mmHg to 79.5 mmHg [12, 13]. The discordant results can be explained by the different locations of injections and measurement methods. In their study on rabbits, Wallyn et al. found an increase in PaO_2_ in the anterior chamber from a baseline value of 63.5 mmHg to a value of 503 mmHg treatment with hyperbaric oxygen [6]. Our baseline value for PaO_2_ was 78.5 mmHg, which was close to the results of Kwan et al. [12]. PaO_2_ significantly increased after the injection of the air or oxygen bubble into the anterior chamber. PaO_2_ in the 50% oxygen injected group was significantly higher compared with that in the air injected group. Even though there was a higher PaO_2_ increase in the 50% oxygen injected group compared to the air bubble injected group, there were one cataract formation and one persistent corneal edema at the end of the 4th week. We believe that injecting 50% oxygen into the anterior chamber significantly increases the free radicals that cause toxic effects on the lens and corneal endothelium. As such, even though this supplies a significant amount of increase in the PaO_2_ of aqueous humour, it is not a safe treatment modality. On the contrary, air bubble injected group did not reveal any corneal or lens pathologies. Additionally, air bubbles are often injected into the anterior chamber in anterior segment surgeries such as descemet membrane detachment or keratoplasty, and this is thought to have no potential side effects. Therefore, we propose that injection of air bubble into the anterior chamber after anterior segment lavage is an easy, safe, and cost effective method.

The limitation of the study is quantification of sickled cells before and after the treatment. In similar previous studies, which aimed to increase the anterior chamber oxygen concentration, it was shown that sickling was decreased after hyperbaric oxygen treatment and IOP was decreased in transcorneal oxygen therapy [6, 10]. As such, we believe those studies support our study in respect of clearance of sickled cells from anterior chamber and trabecular meshwork.

The washout time for hyphema in patients with sickle cell trait lasts longer than in normal patients. Previous researches revealed that the sickled erythrocytes block the trabecular meshwork, thereby decreasing the aqueous outflow which results in increased IOP. IOP increase causes ischemia and increases sickling which triggers a vicious cycle [14, 15]. Friedman et al. described four cases of IOP increases in sickle cell trait patients due to secondary glaucoma; three patients had microscopic hyphema and one had spontaneous haemorrhage in Schlemm's canal [16]. After resorption of blood from the trabecular meshwork and Schlemm's canal, the patients' IOP decreased to normal levels. We think that injection of air into the anterior chamber may provide better drainage of blood from the trabecular meshwork and Schlemm's canal. Using this method, it is likely that IOP elevation can be managed without or at least with smaller doses of medication.

Nasrullah and Kerr reported that sickle cell trait is a significant risk factor for secondary hemorrhage in children with traumatic hyphema [17]. They also found that hyphema patients with sickle cell trait had higher intraocular pressure and permanent vision loss than the other patients [17]. Even small elevations of IOP in patients with sickle cell disease/trait can cause serious complications in the postoperative period; thus, it is crucial to remove the potential erythrocytes obstructing outflow from the anterior chamber, trabecular meshwork, and Schlemm's canal as quickly as possible. In our study, the air bubbles injected into the anterior chamber were absorbed in 8 hours. Therefore, injecting air bubbles into the anterior chambers of patients with sickle cell disease/trait following lavage may wash erythrocytes from the anterior chamber, trabecular meshwork, and Schlemm's canal more rapidly and easily. Consequently, using fewer or no drugs can control the possible postoperative IOP elevation.

## 5. Conclusions

Injection of air bubble into the anterior chamber safely provides an increase in PaO_2_ in aqueous humour. It is likely that this increase will accelerate the removal of sickled erythrocytes from the anterior chamber, trabecular meshwork, and Schlemm's canal with less effort. We offer to leave an air bubble in the anterior chamber of patients with sickle cell hemoglobinopathies and hyphema undergoing an anterior chamber washout.

## Figures and Tables

**Table 1:** Aqueous humour PaO_2_ values.

Groups	PaO_2_ (mmHg)		
	Mean ± SD	Median ± SEM	Range
Group 1	78.45 ± 9.9	78.15 ± 3.5	60.4–90.7
Group 2	73.97 ± 8.86	72.35 ± 3.13	59.2–86.3
Group 3	123.35 ± 13.6	124.60 ± 4.8	101.7–147.5
Group 4	306.47 ± 16.5	304.65 ± 7.77	265.4–340.6

**Table 2:** Comparison of the groups.

Groups	*P* value
1-2	*P* > 0.05
1–3	*P* < 0.001
1–4	*P* < 0.001
2-3	*P* < 0.001
2–4	*P* < 0.001
3-4	*P* < 0.001

## References

[1] Walton W, von Hagen S, Grigorian R, Zarbin M (2002). Management of traumatic hyphema. *Survey of Ophthalmology*.

[2] Cohen SB, Goldberg MF, Fletcher ME, Jednock NJ (1986). Diagnosis and management of ocular complications of sickle hemoglobinopathies. Part V. *Ophthalmic Surgery*.

[3] Sharma A, Ibarra MS, Piltz-Seymour JR, Syed NA (2003). An unusual case of uveitis-glaucoma-hyphema syndrome. *The American Journal of Ophthalmology*.

[4] Goldberg MF, Dizon R, Moses VK (1979). Sickled erythrocytes, hyphema, and secondary glaucoma. VI. The relationship between intracameral blood cells and aqueous humor pH, PO_2_, and PCO_2_. *Ophthalmic Surgery*.

[5] Lange RD, Minnich V, Moore CV (1951). Effect of oxygen tension and of pH on the sickling and mechanical fragility of erythrocytes from patients with sickle cell anemia and the sickle cell trait. *The Journal of Laboratory and Clinical Medicine*.

[6] Wallyn CR, Jampol LM, Goldberg MF, Zanetti CL (1985). The use of hyperbaric oxygen therapy in the treatment of sickle cell hyphema. *Investigative Ophthalmology and Visual Science*.

[7] Goldberg MF (1979). Sickled erythrocytes, hyphema, and secondary glaucoma. I. The diagnosis and treatment of sickled erythrocytes in human hyphemas. *Ophthalmic Surgery*.

[8] Michelson PE, Pfaffenbach D (1972). Retinal arterial occlusion following ocular trauma in youths with sickle-trait hemoglobinopathy. *The American Journal of Ophthalmology*.

[9] Goldberg MF, Dizon R, Raichand M (1979). Sickled erythrocytes, hyphema, and secondary glaucoma. III. Effects of sickle cell and normal human blood samples in rabbit anterior chambers. *Ophthalmic Surgery*.

[10] Benner JD (2000). Transcorneal oxygen therapy for glaucoma associated with sickle cell hyphema. *The American Journal of Ophthalmology*.

[11] Deutsch TA, Weinreb RN, Goldberg MF (1984). Indications for surgical management of hyphema in patients with sickle cell trait. *Archives of Ophthalmology*.

[12] Kwan M, Niinikoski J, Hunt TK (1972). In vivo measurements of oxygen tension in the cornea, aqueous humor, and anterior lens of the open eye. *Investigative ophthalmology*.

[13] Wegener JK, Moller PM (1971). Oxygen tension in the anterior chamber of the rabbit eye. *Acta Ophthalmologica*.

[14] Goldberg MF (1979). The diagnosis and treatment of secondary glaucoma after hyphema in sickle cell patients. *The American Journal of Ophthalmology*.

[15] Goldberg MF (1978). The diagnosis and treatment of sickled erythrocytes in human hyphemas. *Transactions of the American Ophthalmological Society*.

[16] Friedman AH, Halpern BL, Friedberg DN (1979). Transient open-angle glaucoma associated with sickle cell trait: report of 4 cases. *British Journal of Ophthalmology*.

[17] Nasrullah A, Kerr NC (1997). Sickle cell trait as a risk factor for secondary hemorrhage in children with traumatic hyphema. *The American Journal of Ophthalmology*.

